# The effect of total factor productivity of forestry industry on CO_2_ emissions: a spatial econometric analysis of China

**DOI:** 10.1038/s41598-021-93770-z

**Published:** 2021-07-09

**Authors:** Shen Zhong, Hongli Wang

**Affiliations:** School of Finance, Harbin University of Commerce, Heilongjiang, 150000 People’s Republic of China

**Keywords:** Environmental sciences, Environmental social sciences

## Abstract

Forestry plays an essential role in reducing CO_2_ emissions and promoting green and sustainable development. This paper estimates the CO_2_ emissions of 30 provinces in China from 2008 to 2017, and uses Global DEA-Malmquist to measure the total factor productivity of the forestry industry and its decomposition index. On this basis, by constructing a spatial econometric model, this paper aims to empirically study the impact of forestry industry's total factor productivity and its decomposition index on CO_2_ emissions, and further analyze its direct, indirect and total effects. The study finds that the impact of forestry industry's total factor productivity on CO_2_ emissions shows an "inverted U-shaped" curve and the inflection point is 0.9395. The spatial spillover effect of CO_2_ emissions is significantly negative. The increase of CO_2_ emissions in adjacent areas will provide a "negative case" for the region, so that the region can better address its own energy conservation and emission reduction goals. TFP of forestry industry also has positive spatial spillover effect. However, considering the particularity of forestry industry, this effect is not very significant. For other factors, such as foreign direct investment, urbanization level, industrial structure and technology market turnover will also significantly affect regional CO_2_ emissions.

## Introduction

In 2019, the "Trends in Global CO_2_ and Total Greenhouse Gas Emissions: 2019 Report”, issued by the Netherlands Environmental Assessment Agency (PBL), showed that the emissions of global greenhouse gas (GHG) in the past 10 years were increasing at a rate of 1.5% per year, only slowing down from 2014 to 2016. The dramatic increase in greenhouse gas emissions has led to a series of ecological problems, such as the melting of glaciers and rising sea levels, which have seriously endangered the living space of mankind. As an important part of greenhouse gases, CO_2_ has also become a global issue. In 2018, global CO_2_ emissions hit a record high of 33 billion tons. Among them, China's CO_2_ emissions were about 10 billion tons, which accounting for 30%^1^. Since the reform and opening up in 1978, China's economy has developed rapidly and achieved remarkable results. However, the rapid economic development has also brought a lot of energy consumption and environmental problems. In 2007, China has surpassed the United States to become the world's largest CO_2_ emitter, and in 2010, China also surpassed Japan to become the world's second largest economy in 2010. Therefore, the implementation of China's energy conservation and emission reduction policies plays an important role in improving the global ecological environment^2,3^. In order to reduce CO_2_ emissions, China has made many efforts in policy formulation and economic activities. In terms of policy, in 2012, the report of the Eighteenth National Congress of the Communist Party of China raised the construction of ecological civilization to the same status as economic, political, cultural and social development. In 2016, the "Thirteenth Five-Year Plan for Ecological Environmental Protection" also regarded the ecological environmental governance as the key to China's sustainable development, and established a total management system for forests, grasslands, and field. In 2018, the "Three-Year Action Plan for Winning the Blue-Sky Defense" also proposed to accelerate the improvement of environmental air quality and achieve a multi-win situation for environmental, economic and social benefits. In terms of economic activities, the state has also taken a series of measures to promote energy conservation and emission reduction, including the development of new energy industries, such as solar and nuclear energy, the advocacy of "Low-carbon Transportation", the development of "Green Agriculture", and the implementation of "Natural Protection projects."

The "United Nations Framework Convention on Climate Change" puts forward the ultimate goal of maintaining atmospheric greenhouse gas concentrations at a stable level. Therefore, many countries have actively taken measures to reduce CO_2_ emissions and have begun to constantly try carbon sequestration technologies^4,5^. Forestry, as one of the components of China’s national economy, is a production sector that protects the ecological environment, cultivates and protects forests to obtain timber and other forest products, and uses the natural characteristics of forest trees to play a protective role. It plays an important role in reducing emissions and sequestering carbon^6–8^. Koponen et al. pointed out that forestry is a basic industry and social public welfare, and it engaged in cultivating, protecting and utilizing forest resources through advanced science and technology and management means in man and biosphere^9^. By giving full play to the multiple benefits of forest and continuing to manage the forest resources, it also can promote the coordinated development of population, economy, society, environment and resources. Gu et al. found that the carbon storage of China's forests has been increasing, which effectively reduces the CO_2_ content in the atmosphere^10^. Ray and Jana also reached a similar conclusion that forests have the function of carbon sinks and are an important method for managing CO_2_ emissions^11^. Moreover, China has also gradually realized the key role of forestry in reducing CO_2_ emissions, especially its powerful carbon storage function^12^. Poudyal et al. believed that active forest management is necessary to promote sustainable forest management, especially to reduce deforestation and forest degradation and pollutant emissions in developing countries^13^. This was also in line with the research conclusion of Niles et al.^14^. Qiu et al. studied the growth dynamics, biomass and carbon storage of arbor forests, economic forests and shrubs based on the biomass data of 7801 national forest plots and related forest ecosystems in 2003, 2008 and 2013, and they pointed out that the carbon storage, density and carbon sink of China's forest vegetation increased rapidly, and forest carbon storage was mainly concentrated in the southwest and northeast regions, with the highest carbon density in the southwest^15^. It was further predicted that from 2020 to 2050, China’s forest vegetation would absorb 22.14% of the carbon dioxide emissions from the burning of fossil fuels, which will help slow the growth of greenhouse gas emissions in the next 30 years. From the perspective of forest economic value, Deng et al. found that among the strategic methods to reduce carbon emissions, afforestation had the highest economic value^16^. Therefore, the development of the forestry industry is crucial to reducing CO_2_ emissions. There are many indicators to measure the development of the forestry industry, for example, single indicators, such as forest stock and forest area, and multiple indicators, such as input–output efficiency system. However, scholars generally believe that the efficiency system is one of the most reasonable indicators to measure industrial development^17^. Obviously, total factor productivity is the most representative of them. It can represent the part of output that cannot be explained by production input, can effectively measure the utilization efficiency and intensity of input and output, and is a key indicator to measure economic efficiency^18^. Therefore, this article takes the forestry industry's total factor productivity as a proxy variable to measure the development of the forestry industry, and explores its impact on CO_2_ emissions.

China has a vast land and abundant resources, and different regions have different resource endowments. In particular, the key factors that affecting the development of the forestry industry, such as climate, soil, and tree varieties, also have significant regional differences. However, on the one hand, China, as a large country with coordinated management and development, its macroeconomic policy requires each province to develop in differentiated way, at the same time, it should adhere to the red line of forest ecological protection, build a strong ecological security barrier, and consolidate the ecological foundation. On the other hand, with the flow of forestry capital and the popularization of technology, decision makers in various regions refer to each other and formulate policies and guidelines based on the "yardstick", forming a "synergistic effect." The combined effect of the two makes the development of the forestry industry have a certain spatial correlation. Rajashekar et al. found that as an important part of the global carbon cycle, the spatial distribution of forest carbon had a significant impact on the carbon intensity of different regions^19^. Wang et al. indicated that the spatial agglomeration of CO2 emissions in various provinces in China was gradually increasing^20^. Tian and Zhou also reached a similar conclusion and proposed that understanding the spatial effect of CO_2_ was one of the key factors in reducing CO_2_ emissions^21^. Therefore, the spatial dynamics of regional CO_2_ emissions and forestry industry development cannot be ignored.

To sum up, the research of existing literature provides a meaningful reference, however, there are still several deficiencies: Firstly, the existing study mainly focuses on exploring the environmental impact of industry or manufacturing industry, the research on exploring the environmental impact of forestry industry is relative rare; Secondly, the researches on forestry industry mainly focused on the industrial development and the economic benefits of forestry products, rather than the research on industry total factor productivity, especially the impact of forestry industry total factor productivity on CO_2_ emissions. Thirdly, when considering the impact of total factor productivity on CO_2_ emissions, the existing literature took each province as an independent individual, and did not fully consider the impact of spatial factors. Therefore, this paper attempts to explore the impact of total factor productivity of China's forestry industry on regional CO_2_ emissions from a spatial perspective. Specifically, the contribution of this paper may be as follows: Firstly, it uses DEA Global-Malmquist to measure the total factor productivity of forestry industry in 30 provinces of China from 2005 to 2016, and further analyzes its decomposition index. Secondly, it measures the CO_2_ emissions of eight kinds of energy in 30 provinces of China and discusses their spatial spillover effects. Thirdly, it builds a spatial econometric model to empirically study the impact of TFP and its decomposition index on CO2 emissions, and further analyzes its direct effect, indirect effect and total effect.

The arrangement of the paper is as follows: the second part introduces the methods of empirical research, including DEA Global-Malmquist and spatial econometric model; the third part is the data description, including the calculation of total factor efficiency and CO_2_ emissions of forestry industry; the fourth part presents the results and discussion of empirical research; the final part summarizes the research conclusions, and proposes some corresponding countermeasures, shortcomings and future research route of this paper.

## Method

### Malmquist index

Malmquist proposed the calculation method of Malmquist index and applied it to the study of consumption changes in the same period^22^, and Caves et al. applied this index to productivity measurement for the first time^23^. Later, in order to be more widely used to measure productivity changes in a period, the academic community combined the index with the data envelopment analysis method (DEA), proposed by Charens et al.^24^. The specific methods are as follows:

The distance function based on the input angle is:

1 $$ \overrightarrow {{D^{t} }} (x,y) = \min _{\theta } \{ \theta :(\theta x,y) \in p^{t} (x,y),\theta  > 0\} $$

By considering the t period as the based period, the input productivity index can be defined as:

2 $$ M^{t}  = \overrightarrow {{D^{{t + 1}} }} (x^{{t + 1}} ,y^{{t + 1}} )/\overrightarrow {{D^{t} }} (x^{t} ,y^{t} ) $$

Then the productivity index based on (t + 1) can be defined as:

3 $$ M^{{t + 1}}  = \overrightarrow {{D^{{t + 1}} }} (x^{{t + 1}} ,y^{{t + 1}} )/\overrightarrow {{D^{{t + 1}} }} (x^{t} ,y^{t} ) $$

Fare et al. further decomposed the Malmquist index production efficiency into technical efficiency change index (EC) and technical progress efficiency (TC) by constructing the Malmquist index production efficiency model from t period to (t + 1)^25^, the model are as follows:

4 $$ \begin{aligned}    & M(x^{{t + 1}} ,y^{{t + 1}} ,x^{{t + 1}} ,y^{{t + 1}} ) \\     & \quad  = \left\{ {\frac{{\overrightarrow {{D^{{t + 1}} }} (x^{{t + 1}} ,y^{{t + 1}} )}}{{\overrightarrow {{D^{{t + 1}} }} (x^{{t + 1}} ,y^{{t + 1}} )}}\frac{{\overrightarrow {{D^{t} }} (x^{{t + 1}} ,y^{{t + 1}} )}}{{\overrightarrow {{D^{t} }} (x^{t} ,y^{t} )}}} \right\}^{{{\raise0.7ex\hbox{$1$} \!\mathord{\left/ {\vphantom {1 2}}\right.\kern-\nulldelimiterspace} \!\lower0.7ex\hbox{$2$}}}}  \\     & \quad  = \frac{{\overrightarrow {{D^{{t + 1}} }} (x^{{t + 1}} ,y^{{t + 1}} )}}{{\overrightarrow {{D^{t} }} (x^{t} ,y^{t} )}}\left[ {\frac{{\overrightarrow {{D^{t} }} (x^{{t + 1}} ,y^{{t + 1}} )}}{{\overrightarrow {{D^{{t + 1}} }} (x^{{t + 1}} ,y^{{t + 1}} )}}\frac{{\overrightarrow {{D^{t} }} (x^{t} ,y^{t} )}}{{\overrightarrow {{D^{{t + 1}} }} (x^{t} ,y^{t} )}}} \right]^{{{\raise0.7ex\hbox{$1$} \!\mathord{\left/ {\vphantom {1 2}}\right.\kern-\nulldelimiterspace} \!\lower0.7ex\hbox{$2$}}}}  \\     & \quad  = EC*TC \\  \end{aligned} $$

Among them, $\overrightarrow {{D^{t} }} (x^{t} ,y^{t} )$ and $\overrightarrow {{D^{t} }} (x^{{t + 1}} ,y^{{t + 1}} )$ represents the technical efficiency level at period t and period (t + 1) when using the technology of period t as a benchmark. And $\overrightarrow {{D^{{t + 1}} }} (x^{t} ,y^{t} )$ and $\overrightarrow {{D^{{t + 1}} }} (x^{{t + 1}} ,y^{{t + 1}} )$ is the technology efficiency level at period t and period (t + 1) when using the technology of period (t + 1) as a benchmark. If $M > 1$, the productivity from period t to period (t + 1) will increase; otherwise, it will decrease. Furthermore, the index is further decomposed into EC and TC, among them, the change in technical efficiency (EC), with constant return to scale, measures the catch-up angle from period t to period (t + 1) for each observed object to the best practice boundary, and the technical progress efficiency (TC), with constant return to scale, measures the movement of technology boundary from t period to (t + 1) period.

### Global-Malmquist index

However, Pastor and Lovell put forward three defects in the traditional Malmquist index^26^: Firstly, the geometric mean calculation method does not formally satisfy the transmissibility; Secondly, linear programming may not have a feasible solution, if the technology is not referred to in the same period. Thirdly, traditional Malmquist index methods usually show more frequent technical retrogression in estimating environmental performance. The defects in these three aspects lead to the fact that the measurement of traditional ML productivity index may mislead decision makers. Therefore, in order to overcome the problems existing in traditional ML indexes, Pastor and Lovell proposed the Global Malmquist Index. It is obvious that the Global Malmquist index improves the definition of the production probability set, it not only defines the production technology set of the same period, but also defines a global production technology set that includes all observation units and reference technology sets for all periods. In the whole research period, using only one global production technology set to avoid the defects that cannot meet the transitivity due to the form of traditional Malmquist exponential geometric average. This method has been widely used^27,28^. Its decomposition index is:

5 $$ \begin{aligned}   TFP^{G}  &  = M^{G} (x^{{t + 1}} ,y^{{t + 1}} ,x^{t} ,y^{t} ) = \frac{{\overrightarrow {{D^{G} }} (x^{{t + 1}} ,y^{{t + 1}} )}}{{\overrightarrow {{D^{G} }} (x^{t} ,y^{t} )}} \\     & \quad  = \frac{{\overrightarrow {{D^{{t + 1}} }} (x^{{t + 1}} ,y^{{t + 1}} )}}{{\overrightarrow {{D^{t} }} (x^{t} ,y^{t} )}}*\left\{ {\frac{{\overrightarrow {{D^{G} }} (x^{{t + 1}} ,y^{{t + 1}} )}}{{\overrightarrow {{D^{{t + 1}} }} (x^{{t + 1}} ,y^{{t + 1}} )}}*\frac{{\overrightarrow {{D^{t} }} (x^{t} ,y^{t} )}}{{\overrightarrow {{D^{G} }} (x^{t} ,y^{t} )}}} \right\} \\     & \quad  = \frac{{\overrightarrow {{D^{{t + 1}} }} (x^{{t + 1}} ,y^{{t + 1}} )}}{{\overrightarrow {{D^{t} }} (x^{t} ,y^{t} )}}*\left\{ {\frac{{\overrightarrow {{D^{G} }} (x^{{t + 1}} *\overrightarrow {{D^{{t + 1}} }} (x^{{t + 1}} ,y^{{t + 1}} ),y^{{t + 1}} )}}{{\overrightarrow {{D^{G} }} (x^{t} *\overrightarrow {{D^{t} }} (x^{t} ,y^{t} ),y^{t} }}} \right\} \\     & \quad  = EC*TC \\  \end{aligned} $$

The meaning of the symbol is the same as (4).

### Spatial autocorrelation test

#### Global Moran’s Index

A series of methods for measuring spatial correlation have been proposed in academic circles, among which the most popular one is "Moran index I"^29^:

6 $$ Moran^{\prime}s{\mkern 1mu} I_{{it}}  = \frac{1}{{\sum\nolimits_{{i = 1}}^{n} {\sum\nolimits_{{j = 1}}^{n} {w_{{ij}} } } }} \times \frac{{\sum\nolimits_{{i = 1}}^{n} {\sum\nolimits_{{j = 1}}^{n} {w_{{ij}} (x_{{it}}  - \bar{x})(x_{{jt}}  - \bar{x}} )} }}{{\sum\nolimits_{{i = 1}}^{n} {(x_{{it}}  - \bar{x})^{2} /n} }} $$

where $\bar{x} = \frac{1}{n}\sum\nolimits_{{i = 1}}^{n} {x_{{it}} }$, $x_{{it}}$ is the sample for the *i*th province at the t year. $W_{{ij}}$ represents the distance between region i and j, representing the (i, j) elements in the spatial weight matrix, $\sum\nolimits_{{i = 1}}^{n} {\sum\nolimits_{{j = 1}}^{n} {w_{{ij}} } }$ is the sum of all elements of spatial weight. The value of Moran’s Index is generally between − 1 and 1, and if MI > 0, it means that variables are positively correlated in space, namely, high value and high value aggregate together (low value and low value aggregate together); Conversely, if MI < 0, it indicates that the high value and the low value are adjacent; MI = 0 denotes that there is no spatial correlation, that is, the distribution of high value and low value are completely random.

#### Geary’s C

Besides MI, Geary index C (Geary's C) is another index to examine the spatial autocorrelation 30, it is also called the Geary adjacent Ratio (Geary's Contiguity ratio):

7 $$ C = \frac{{(n - 1)\sum\nolimits_{{i = 1}}^{n} {\sum\nolimits_{{j = 1}}^{n} {w_{{ij}} (x_{i}  - x_{j} )^{2} } } }}{{2\left( {\sum\nolimits_{{i = 1}}^{n} {\sum\nolimits_{{j = 1}}^{n} {w_{{ij}} } } } \right)\left[ {\sum\nolimits_{{i = 1}}^{n} {(x_{i}  - \bar{x})^{2} } } \right]}} $$

The value of Geary's C is generally between 0 and 2, and changes inversely with MI. Where $C > 1$ means there is a negative correlation in space,$C = 1$ means there is no correlation, and $C < 1$ represents a positive correlation.

#### Local Moran’s index

Considering that "Global Moran's I" is to investigate the spatial agglomeration of the whole spatial sequence, in order to further analyze the spatial agglomeration of a certain region, this paper introduces "Local Moran's I":

8 $$ Moran^{\prime}s{\mkern 1mu} I_{{it}}  = \frac{{(x_{{it}}  - \bar{x})\sum\nolimits_{{j = 1}}^{n} {W_{{ij}} } (x_{{jt}}  - \bar{x})}}{{\sum\nolimits_{{i = 1}}^{n} {(x_{i}  - \bar{x})^{2} } /n}} $$

The meanings of all symbols in Eq. (8) are consistent with that in Eq. (6).

### Spatial panel model

Spatial economics suggests that every thing is not an independent individual, and there is a spatial correlation between things^31^. This paper constructs three spatial econometric models respectively, including Spatial Autoregressive Model (SAR), Spatial Error Model (SEM) and Spatial Durbin Model (SDM). Compared with SAR and SEM, SDM model contains endogenous and exogenous interaction effects, and it examines the influence of the explained variables of the region on its neighbor independent variables. The general form of the three methods is as follows:

SEM:

9 $$ y = X\beta  + \mu $$

10 $$ \mu  = \rho W\mu  + \varepsilon ,\varepsilon \sim N(0,\sigma ^{2} I_{n} ) $$

SAR:

11 $$ y = X\beta {\text{ + }}\lambda Wy + \varepsilon $$

SDM:

12 $$ y = X\beta  + \lambda Wy + \theta WX + \varepsilon $$

where $\beta$ is the coefficient of the independent variable, and $W$ is spatial weight matrix.$\theta$ refers to the spatial lag coefficient of the independent variable, indicating the effect degree by the independent variable in surrounding regions on the dependent variable in the local region.$\lambda$ stands for the spatial autoregressive coefficient, which denotes the impact effect of dependent variable’s spatial dependence.$\varepsilon$ and $\mu$ refers to the error term and disturbing term respectively.

### Direct effect, indirect effect and total effect

When exploring spatial effects, LeSage & Pace proposed that the research should be conducted from three aspects: direct effects, indirect effects and total effects^32^. Therefore, in order to comprehensively analyze all the impact paths of TFP in the forestry industry on CO2 emissions, this paper adopts the partial differential equations to divide the comprehensive influence of TFP on CO2 emissions into direct effects and indirect effects. The direct effect refers to the influence of the change of the explanatory variable on the explained variable in the local region; the indirect effect, also called the spillover effect, measures the degree of the influence of the change of the explanatory variable in the local region on the explained variable in other regions, and the total effect is the sum of direct and indirect effect. The specific calculation methods of direct effects, indirect effects and total effects based on the spatial Dubin model are as follows:

13 $$ Y = (I - \rho W)^{{ - 1}} n\ell _{n}  + (I - \rho W)^{{ - 1}} (X\beta  + WX\gamma ) + AZ + (I - \rho W)^{{ - 1}} \varepsilon $$

By obtaining the partial differential equation of the explained variable vector Y in the general form of the spatial Durbin model of Eq. (13) on the k-th explanatory variable, we can obtain the following partial derivative matrix, as shown in the following equation:

14 $$ \left( {\frac{{\partial Y}}{{\partial X_{{1k}} }}{\text{ }}\frac{{\partial Y}}{{\partial X_{{2k}} }}{\text{ }} \cdots {\text{ }}\frac{{\partial Y}}{{\partial X_{{nk}} }}} \right) = \left[ {\begin{array}{*{20}c}    {\frac{{\partial Y_{1} }}{{\partial X_{{1k}} }}} &  \cdots  & {\frac{{\partial Y_{1} }}{{\partial X_{{nk}} }}}  \\     \vdots  &  \ddots  &  \vdots   \\    {\frac{{\partial Y_{n} }}{{\partial X_{{1k}} }}} &  \cdots  & {\frac{{\partial Y_{n} }}{{\partial X_{{nk}} }}}  \\   \end{array} } \right] = (I - \rho W)^{{ - 1}} \left[ {\begin{array}{*{20}c}    {\beta _{k} } & {\omega _{{12}} \gamma _{k} } &  \cdots  & {\omega _{{1n}} \gamma _{k} }  \\    {\omega _{{21}} \gamma _{k} } & {\beta _{k} } &  \cdots  & {\omega _{{2n}} \gamma _{k} }  \\     \vdots  &  \vdots  &  \ddots  &  \vdots   \\    {\omega _{{n1}} \gamma _{k} } & {\omega _{{n2}} \gamma _{k} } &  \cdots  & {\beta _{k} }  \\   \end{array} } \right] $$

In Eq. (14), the direct effect of the k-th explanatory variable is the average value of each element of the main diagonal in the matrix, and the indirect effect of the k-th explanatory variable is the average value of all elements in the matrix except the elements of main diagonal.

## Variables and data

### Dependent variable

According to the standard coal method provided by the "2006 IPCC National Greenhouse Gas Inventory Guidelines" (Table 1), this paper calculates the carbon emissions of 8 energy consumption (coal, coke, crude oil, gasoline, kerosene, diesel, natural gas and fuel oil) in 30 provinces in China from 2006 to 2016, the specific formula is as follows:

15 $$ CO_{{2ti}}  = \sum\limits_{{j = 1}}^{8} {CO_{{2tij}}  = } \sum\limits_{{i = 1}}^{8} {E_{{tij}} *NCV_{j} *CEF_{j} *COF*44/12} $$

where *t*, *i* and *j* represents the year, province and energy respectively; $CO_{{2ti}}$ denotes CO_2_ emissions of the *i*th province in *t* year, $E_{{tij}}$ represents the consumption of *j* energy for *i* province in *t* year; $NCV_{j}$ refers to the net calorific value of the energy of *j*, $CEF_{j}$ is carbon emission factor for the energy of *j*, and the $COF$ is the carbon oxidation coefficient. In addition, considering that when carbon is oxidized to CO_2_, the molecular weight changes from 12 to 44. Therefore, the corresponding conversion is performed when calculating CO_2_ emissions.

**Table 1:** Carbon emission coefficients of various energy. Data source: IPCC (2006) and Energy research institute of National Development and Reform Commission (2007).

Energy category	Lower calorific value $F_{j}$ (kJ/kg)	Carbon emission factor $C_{j}$ (tC/TJ)	Carbon oxidation factor	Carbon emission coefficient (gc/Kgce)
Coal	20,908	26.4	0.94	1.9003
Coke	28,435	29.5	0.93	2.8604
Crude Oil	41,816	20.1	0.98	3.0202
Fuel Oil	41,816	21.1	0.98	3.1705
Kerosene	43,070	19.5	0.98	3.0179
Gasoline	43,070	18.9	0.98	2.9251
Natural Gas	38,931	15.3	0.99	2.1622
Diesel	42,652	20.2	0.98	3.0959

### Independent variable

The input indicators of total factor productivity are generally measured from two aspects: human input and capital input. Taking into account the particularity of the forestry industry, this paper adds natural resource elements to the input indicators. In addition, in terms of human input, this paper selects the number of employees in the forestry industry and the area of forest land as the indicator of manpower and natural resource input respectively. In terms of capital investment, many scholars use the amount of forestry fixed asset investment to express. However, as a flow indicator, fixed assets investment affect the current industrial development, but also affect the later output due to the cumulative effect. Therefore, this paper uses the perpetual inventory method^33^ to calculate the forestry capital stock. The specific formula is:

16 $$ K_{t}  = (1 - \tau )K_{{t - 1}}  + I_{t} $$

where $K_{t}$ and $K_{{t - 1}}$ denotes the capital stock in period t and (t − 1); $I_{t}$ represents the real investment in fixed asset in period *t*, and $\tau$ is the depreciation rate. The key step in measuring the capital stock of each period is to determine the capital stock of the base period ($K_{0}$), and the specific calculation formula is:

17 $$ K_{0}  = E_{0} /(g + \tau ) $$

where $K_{0}$ is the capital stock in the base period; $E_{0}$ refers to the actual fixed asset investment completed in the base period; *g* is the arithmetic average of the growth rate of fixed asset investment completed in each period.

This paper selects the total output value of the forestry industry as the representative index of forestry economic output. The total output value of the forestry system industry, including the total output value of the forestry primary, secondary and tertiary industries (such as agriculture, forestry, animal husbandry, fishery, construction, and social service industries), which have a good comprehensive representation in reflecting the total performance and scale of forestry production in each period. Moreover, the forest stock volume not only represents the total scale and level of forest resources in the region, but also reflects the abundance of forest resources and a basic indicator of the quality of the forest ecological environment. Therefore, this paper uses forest stock as one of the output indicators.

### Control variables

Per capita GDP (HGDP): Per capita GDP is the most representative indicator to measure the overall level of regional economic activities. Yi pointed out that the higher the per capita GDP level, the larger the market size of the region, which can provide a sufficient market for the economic growth and development of industrial scale^34^. However, the expansion of the market scale is accompanied by major energy consumption, and also has an impact on CO2 emissions.

Per capital foreign direct investment (FDI): FDI is one of the important driving forces of China's economic development^35^. It is obvious that introducing foreign investment to promote economic development will also have an impact on environmental quality. On the one hand, the “pollution haven hypothesis” proposed by Walter & Ugelow believes that developed countries transfer industries with serious environmental pollution to developing countries through FDI^36^; On the other hand, "pollution halo hypothesis" believes that technology and talent can be spilled from developed countries to developing countries through FDI, and promote the development of developing countries^37^. Hence, the effect of FDI on environmental quality is worth studying.

Technology market turnover (VOL): Innovation in energy-saving and emission-reduction technologies is a crucial way to reduce CO_2_ emissions, and a perfect technology market environment is also an effective guarantee for promoting technological innovation. Therefore, this article uses the technology market turnover to reflect the regional technology market level. The higher the value, the more conducive to the development and use of energy-saving emission reduction technologies.

Urbanization level (URB): The level of urbanization is one of the main factors contributing to the increase in CO_2_ emissions^38^. On the one hand, the region with higher the level of urbanization, due to the large concentration of population, will significantly increase the demand for housing, cars, electricity, etc., thus resulting in a large amount of CO_2_ emissions; on the other hand, the increase in the level of urbanization also improves the service efficiency of public resources, thus reducing CO_2_ emissions. In this paper, the urban population at the end of the year divided by the total population at the end of the year is regarded as a proxy variable for the level of urbanization. The specific calculation method are described as Table 2:

**Table 2:** Calculation method.

Variables	Symbol	Definition	Unit
CO_2_ emissions	CO_2_	Equation (15)	10,000 tons
Total factor productivity	TFP	Equation (5)	
	TFP^2^		
Technical efficiency changes	EC	Equation (5)	
	EC^2^		
Technological progress efficiency	TC	Equation (5)	
	TC^2^		
Openness	FDI	FDI/GDP	%
Technology market	VOL	Technology market turnover	100 million yuan
Economic development level	HGDP	GDP/Total population at the end of the year	100 million yuan/per
The level of urbanization	URB	Year-end urban population/year-end total population	%

### Data source

**Figure 1:**
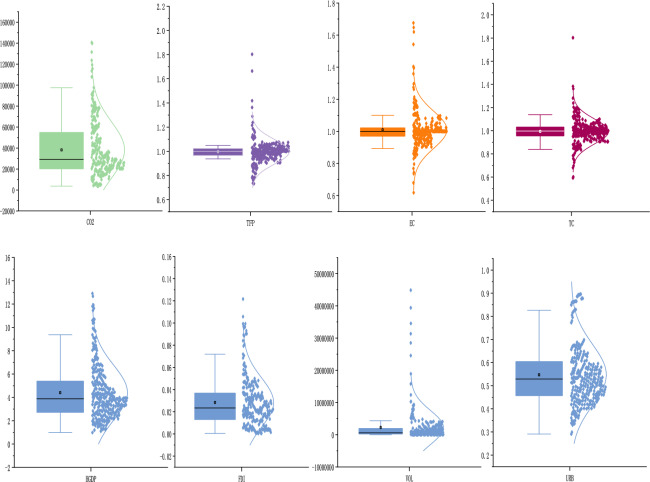
Box plot of variables.

This paper takes 30 provinces in mainland China from 2005 to 2016 (excluding Tibet with incomplete data) as the research object. The data comes from the "China Statistical Yearbook", "China Environmental Statistical Yearbook", "China Forestry Statistical Yearbook", "China Energy Statistical Yearbook". Among them, all monetary indicators are deflated by using the fixed asset investment price index and consumer price index with 2000 as the base year. According to the Table 3 and Fig. 1, the original values of FDI, HGDP, VOL and URB basically accord with the normal distribution characteristics. However, the original values of CO_2_ and HGDP are skewed distribution because some values deviate from the normal distribution and the data are mainly skewed to the lower values. Moreover, the original values of URB, FDI and VOL have singular values. Hence, to make the data more stable and reduce the effect of heteroscedasticity, we logarithmically treat the CO_2_, HGDP, FDI, URB and VOL in this paper.

**Table 3:** Data description.

Variable	Obs	Mean	Std	Min	Max
CO2	300	38,171.27	26,703.55	3618.38	140,602.20
TFP	300	0.996	0.102	0.733	1.803
EC	300	1.010	0.116	0.618	1.676
TC	300	0.994	0.108	0.592	1.803
URB	300	0.547	0.132	0.291	0.896
FDI	300	0.028	0.022	0.000	0.122
SEC	300	4.409	2.357	0.990	12.906
VOL	300	2,257,665	5,270,284	5556.27	4.49e + 07

## Empirical results

### Spatial and temporal characteristics of the CO_2_ emissions distribution

As shown in Table 4, the average CO_2_ emissions of 30 provinces in China from 2008 to 2017 is 3.19 million tons, and the CO_2_ emissions of Hebei, Shanxi, Inner Mongolia, Shandong, Jiangsu, Zhejiang, Henan, Guangdong, and Shaanxi have exceeded the national average. In particular, Shandong province has the highest average CO_2_ emissions, reaching 1.18 million tons, followed by Hebei, Shanxi, Inner Mongolia, Jiangsu, Henan, and Guangdong with average CO_2_ emissions are all over 5 million tons, and Hainan and Qinghai have the lowest value with less than 0.6 million tons.

**Table 4:** CO_2_ emissions in 30 provinces in China from 2008 to 2017.

	2008	2009	2010	2011	2012	2013	2014	2015	2016	2017	Mean
Beijing	12,175.276	12,347.806	12,426.061	11,515.778	11,367.670	9993.187	10,122.876	9106.770	8162.354	7912.467	10,513.024
Tianjin	13,724.624	14,756.765	17,990.527	19,724.019	19,750.233	20,241.108	19,361.792	18,606.495	17,417.360	17,099.883	17,867.281
Hebei	70,277.503	74,870.670	80,454.878	90,953.782	92,051.758	92,046.784	87,362.977	86,254.567	86,372.665	83,922.888	84,456.847
Shaanxi	62,997.116	62,266.688	66,522.157	73,325.741	76,489.895	78,228.238	80,049.015	79,002.950	76,615.820	90,048.674	74,554.629
Neimeng	49,862.954	54,070.174	59,662.904	74,766.073	77,777.615	75,794.784	77,669.163	77,564.365	78,285.737	82,224.944	70,767.871
Liaoning	59,068.697	61,176.937	67,025.751	71,235.365	73,373.227	69,715.929	69,667.728	68,352.776	68,726.665	70,589.915	67,893.299
Jilin	21,745.048	22,197.179	24,702.074	28,374.244	27,969.604	26,763.235	26,558.259	24,824.096	24,271.959	24,205.050	25,161.075
Heiljiang	29,708.116	31,078.559	33,753.927	36,243.641	37,934.543	35,422.782	35,917.502	35,728.426	36,468.733	36,605.250	34,886.148
Shanghai	24,148.082	23,976.646	26,074.620	26,663.720	26,108.077	27,196.491	24,624.418	25,369.346	25,368.881	25,854.732	25,538.501
Jiangsu	56,446.159	59,056.427	65,944.602	75,793.467	77,067.157	78,523.150	77,933.508	80,117.774	83,335.109	81,308.776	73,552.613
Zhejiang	38,207.008	39,710.601	42,422.180	44,739.955	43,318.269	43,274.488	42,402.837	42,879.020	42,379.307	44,250.183	42,358.385
Anhui	26,801.066	29,459.872	31,184.002	33,753.358	34,756.250	37,462.946	38,565.614	38,621.170	38,502.454	39,722.008	34,882.874
Fujian	17,237.552	20,288.312	22,164.263	25,217.565	25,032.012	24,043.000	27,588.817	26,629.461	24,860.410	26,429.744	23,949.114
Jiangxi	14,147.493	14,811.557	17,226.556	18,983.885	19,017.237	20,275.453	20,599.298	21,471.274	21,739.219	22,163.540	19,043.551
Shandong	93,797.854	97,590.729	107,797.004	113,474.261	119,179.294	115,570.869	123,622.690	131,477.812	140,602.191	139,666.803	118,277.951
Henan	54,315.531	55,458.464	59,953.526	66,025.507	61,362.292	60,236.360	60,915.700	60,984.045	60,227.718	56,900.934	59,638.008
Hubei	29,132.857	31,252.978	35,810.121	40,699.272	40,653.448	34,822.074	35,051.602	34,729.986	34,770.222	35,454.198	35,237.676
Hunan	26,146.624	27,464.666	29,128.571	32,464.127	31,932.928	30,691.369	29,714.343	31,264.908	31,478.250	33,021.448	30,330.724
Guangdong	47,168.979	49,843.705	56,535.774	60,761.333	59,693.194	58,830.359	58,935.962	58,841.869	60,447.210	63,138.781	57,419.717
Guangxi	12,750.893	14,138.951	17,195.098	21,135.531	23,204.762	22,887.644	22,714.406	21,570.039	22,680.138	23,851.942	20,212.940
Hainan	4137.225	4468.246	4829.604	5426.186	5724.497	5236.195	5851.927	6534.810	6419.421	6225.887	5485.400
Chongqing	12,314.704	13,298.417	14,644.883	16,734.321	16,321.113	13,877.753	14,773.755	14,818.548	14,608.884	14,641.268	14,603.365
Sichuan	27,738.078	31,134.130	31,233.891	31,933.480	33,443.561	34,229.775	35,215.242	32,741.416	31,327.811	29,785.699	31,878.308
Guizhou	20,970.086	22,991.517	23,173.442	25,616.182	28,030.152	29,010.920	27,956.852	27,693.755	29,359.668	28,913.842	26,371.642
Yunnan	20,818.030	22,624.689	23,905.386	24,671.457	25,626.254	25,230.652	22,634.714	20,203.102	19,991.455	19,695.904	22,540.164
Sxi	25,658.487	27,998.888	33,158.552	36,718.462	42,272.899	44,805.883	47,209.846	46,610.951	47,698.300	48,558.781	40,069.105
Gansu	15,426.467	15,221.593	16,938.379	19,584.152	20,086.192	20,710.679	20,823.488	20,193.677	19,457.217	19,591.572	18,803.342
Qinghai	3618.380	3661.167	3669.063	4258.084	5040.537	5573.429	5161.812	4630.260	5501.526	5165.670	4627.993
Ningxia	9789.090	10,769.053	12,716.216	16,971.112	18,224.748	19,421.096	19,823.883	20,475.744	20,292.350	25,134.550	17,361.784
Xinjiang	19,778.265	23,352.910	26,078.076	30,845.969	35,787.926	40,623.547	44,636.604	45,807.703	49,024.804	52,612.470	36,854.827
Mean	30,670.275	32,377.943	35,477.403	39,287.001	40,286.578	40,024.673	40,448.888	40,436.904	40,879.795	41,823.260	38,171.272

**Figure 2:**
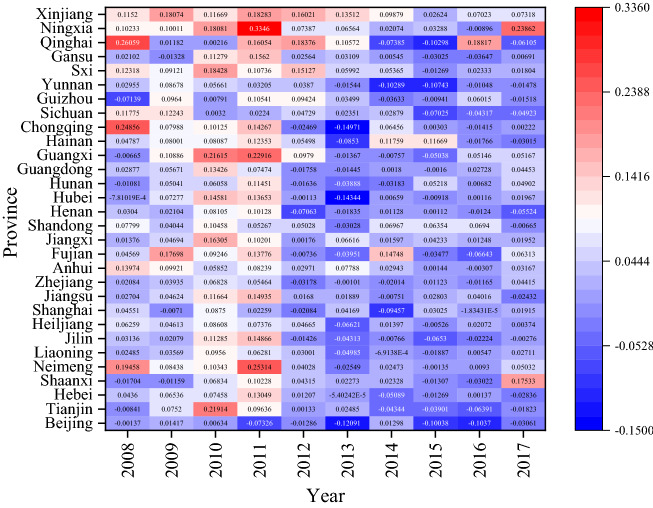
Growth rate of CO_2_ emissions in 30 provinces in China from 2008 to 2017. *Source*: Origin 2021 https://www.originlab.com/doc/.

**Figure 3:**
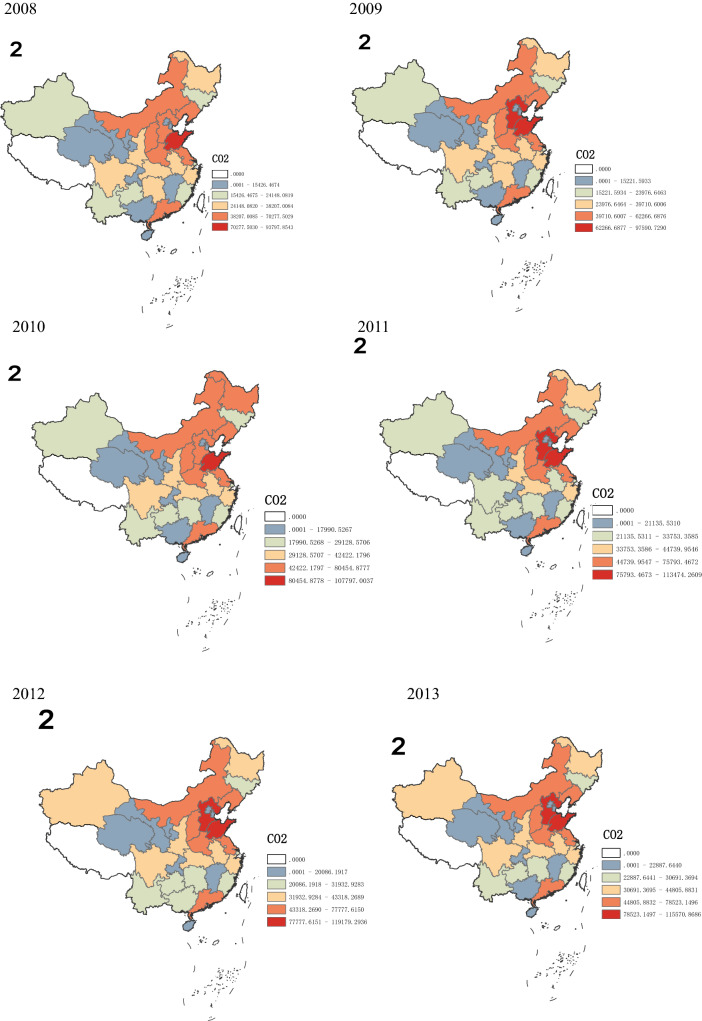

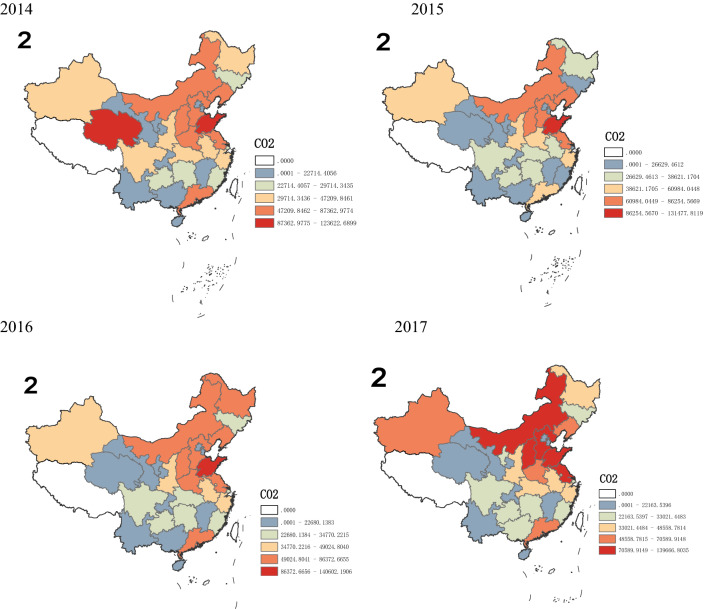
Spatial pattern of China’s emissions. *Source*: ArcMap10.7 https://desktop.arcgis.com/zh-cn/arcmap/.

According to Figs. 2 and 3, from a spatial point of view, there are significant differences in CO_2_ emissions in various regions. From 2008 to 2012, it is obvious that CO_2_ emissions in the eastern region increase significantly, this may be due to the eastern region experiences the fastest economic development with a large number of people gathered and many economic activities took place, thus resulting in a dramatic increase in CO_2_ emissions. The central and western region in China have a relatively slow growth in CO_2_ emissions, because of its relatively stable economic development and population size. After 2012, the growth rate of CO_2_ emissions across the country has dropped significantly, with negative growth in some regions, such as Beijing, Hebei, Liaoning, Jiangsu, and Zhejiang. This may be due to the fact that provinces in the eastern region no longer focus solely on the rapid growth of GDP after 2012, but begins to optimize the industrial structure and promote stable economic development. Especially after 2016, in order to achieve the goals of sustainable development, the eastern region further optimizes the industrial structure, at the same time, the state has proposed a development strategy for the central and western to promote their economic development. Therefore, in recent years, the gap in CO_2_ emissions among different provinces across the country has gradually decreased.

### Analysis of TFP and its decomposition index

**Figure 4:**
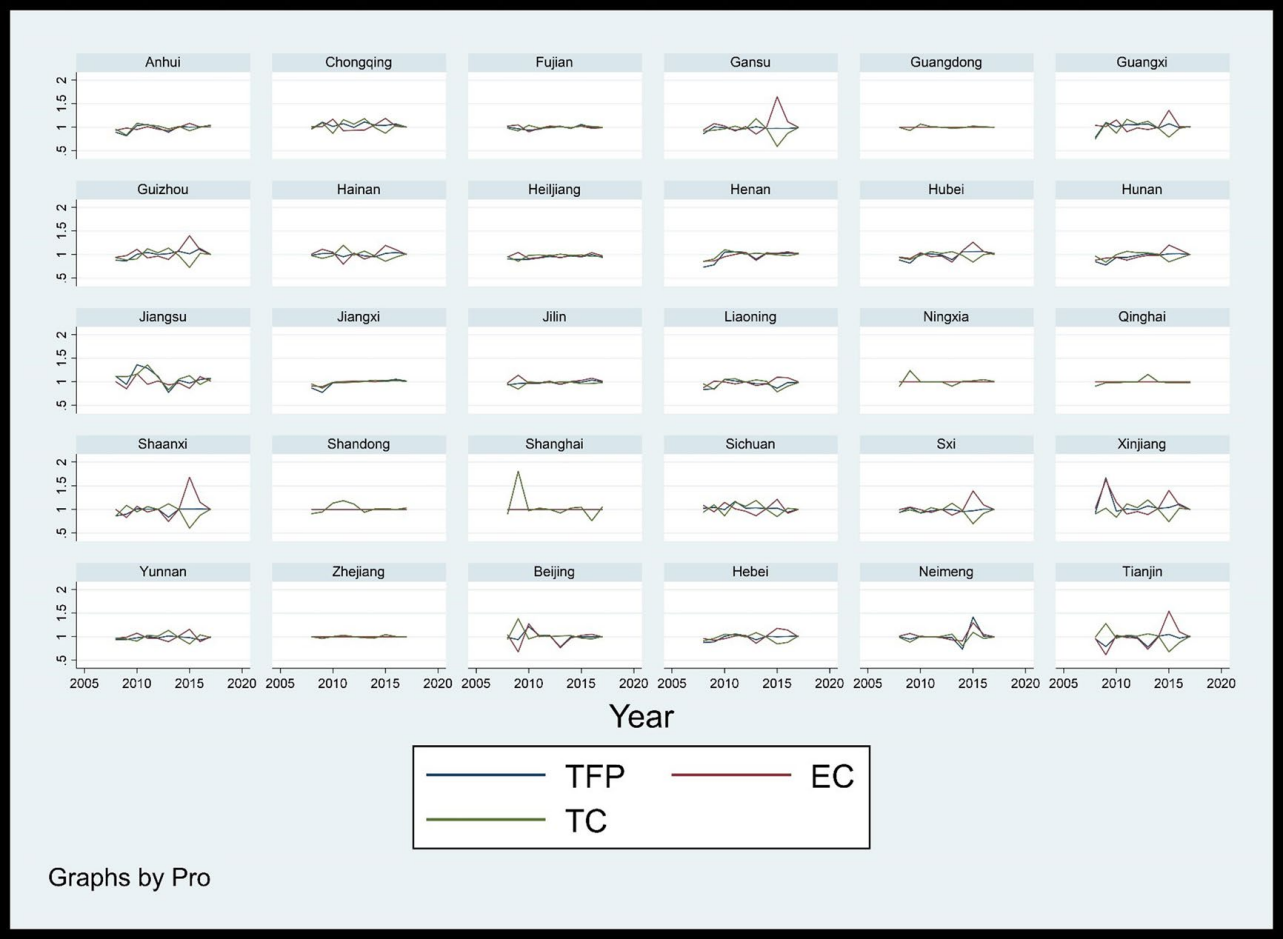
Change trend of TFP and decomposition indexes of forestry industry.

**Figure 5:**
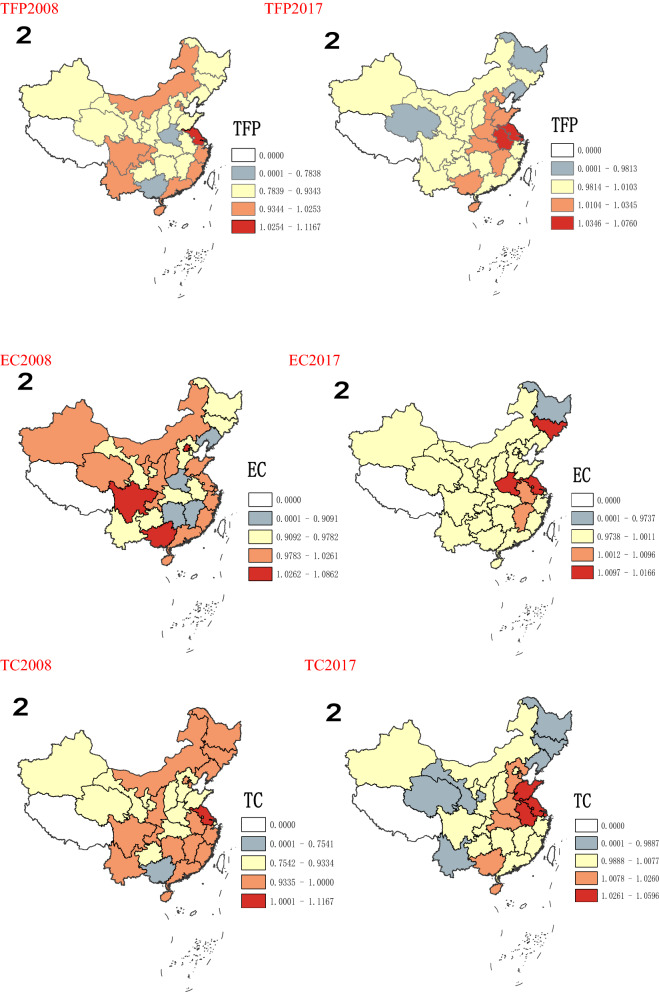
Spatial distribution of TFP, EC, TC. *Source*: ArcMap10.7 https://desktop.arcgis.com/zh-cn/arcmap/.

As shown in Table 5, from 2008 to 2017, the overall level of TFP in China's forestry industry is not high, with an average value of 0.996. Specifically, from the changes of TFP in each period, except for decline in 2008 and 2013, the TFP in other periods are greater than 1. The TFP of forestry industry has the lowest value in 2008, at only 0.922. Moreover, it starts to rise after 2009 and reaches its peak in 2010 at 1.009. This may be affected by the global financial crisis in 2008, and it shows some phenomenons, such as the decline in output of the forestry industry, reduced employment, hindered product exports, accumulation of inventory, and decline in profits. In response to the challenge of the financial crisis, the country has begun to increase investment in the forestry industry, expand domestic demand, further improve the forest management system, and continuously improve the management level of forest land, which enhances the ability to guarantee the forestry industry. Therefore, the TFP of forestry industry rebound rapidly after 2009, especially in Beijing, Shanghai, Tianjin, and Xinjiang (Fig. 4). In addition, the TFP of China's forestry industry experiences a short period of negative growth and then increased again from 2012 to 2013, and then maintains a steady growth, which shows China's forestry industry is moving in the direction of sustainable development. In terms of the average value, from 2008 to 2017, the average TFP of forestry industry in half of China's provinces is less than 1, including Anhui (0.984), Hebei (0.990), Tianjin (0.982), Fujian (0.995), Heilongjiang (0.959), Henan (0.978), Hubei (0.990), Hunan (0.969), Jiangxi (0.983), Jilin (0.988), Liaoning (0.968), Yunnan (0.986), Shaanxi (0.989), Gansu (0.990) Qinghai (0.997), Sxi (0.991). The annual growth rate of TFP in Shanghai is the largest, reaching more than 6%, followed by Inner Mongolia and Xinjiang. As shown in Table 5, from 2008 to 2017, the decomposition indexes of TFP for China's forestry industry (Technical efficiency changes EC, Technical progress efficiency TC) have volatility changes. In fact, the changing trend of TC is more consistent with the changing trend of TFP, and its fluctuations is significantly larger than TFP, indicating that although changes in EC will affect the changes in TFP, TC has a greater impact on TFP. Furthermore, the average value of EC for national forestry industry is 1.010, and there are only 9 provinces with EC value higher than the average, including Chongqing (1.026), Gansu (1.060), Guangxi (1.042), Guizhou (1.041), Hainan (1.018), Jilin (1.016), Shaanxi (1.033), Inner Mongolia (1.026), Xinjiang (1.106). In these provinces, Xinjiang has the highest EC growth rate, more than 3%, and Heilongjiang has negative productivity growth. Moreover, the average value of national forestry industry TC is 0.994. Among them, Jiangsu Province had the highest TC (1.087), followed by Chongqing, Shandong and Shanghai, and Anhui province have the lowest, showing negative growth. As shown in Fig. 5, there are significant spatial differences in TFP and its decomposition index (EC, TC) of forestry industry in each province. As shown in Fig. 5, there are spatial differences existed in forestry industry TFP and its decomposition indexes (EC, TC) in each province. From 2008 to 2017, areas with higher forestry industry TFP gradually shift from eastern China to central and western. EC also has similar characteristics, while regions with high TC still mainly concentrate in eastern region, such as Shanghai, Jiangsu and Fujian. It also further shows that the developed economic level and high-tech talents in the eastern are conducive to promoting technological progress. In addition to giving full play to its own resource advantages, promoting technological progress in the forestry industry in the central and western is also a key factor in its industrial development.

**Table 5:** TFP and decomposition indexes of forestry industry.

	2008			2010			2012			2014			2016			2017		
	TFP	EC	TC	TFP	EC	TC	TFP	EC	TC	TFP	EC	TC	TFP	EC	TC	TFP	EC	TC
Beijing	1.018	0.997	1.021	1.132	1.080	1.048	1.030	1.011	1.018	0.961	0.925	1.039	1.011	1.063	0.951	1.008	0.997	1.011
Tianjin	0.984	1.000	0.984	1.531	1.000	1.531	0.887	1.000	0.887	1.000	1.000	1.000	0.949	1.000	0.949	1.003	1.000	1.003
Hebei	0.963	1.000	0.963	1.008	1.000	1.008	1.019	1.002	1.016	1.007	0.993	1.014	1.005	1.090	0.921	1.010	1.001	1.009
Shaanxi	1.002	1.000	1.002	1.008	1.000	1.008	1.005	1.000	1.005	1.006	1.001	1.005	1.006	1.072	0.938	1.004	1.000	1.004
Neimeng	1.000	1.000	1.000	1.000	1.000	1.000	0.999	1.000	0.999	1.000	1.000	1.000	1.000	1.000	1.000	1.000	1.000	1.000
Liaoning	0.879	0.953	0.922	1.034	1.022	1.012	1.022	0.990	1.033	0.979	1.014	0.965	0.996	1.026	0.971	1.005	0.999	1.006
Jilin	0.959	0.845	1.134	1.067	1.071	0.997	1.059	0.987	1.073	1.029	0.981	1.049	0.976	0.962	1.015	1.020	0.998	1.022
Heiljiang	0.819	0.927	0.884	1.028	1.044	0.985	1.033	0.994	1.039	1.009	0.983	1.026	0.993	0.984	1.009	1.008	0.996	1.011
Shanghai	1.000	1.000	1.000	0.979	1.000	0.979	1.000	1.000	1.000	0.998	1.000	0.998	0.796	1.000	0.796	1.045	1.000	1.045
Jiangsu	1.188	1.000	1.188	1.350	1.000	1.350	1.110	1.000	1.110	1.053	1.014	1.038	1.049	1.042	1.007	1.076	1.017	1.058
Zhejiang	1.000	1.000	1.000	1.005	1.000	1.005	1.000	1.000	1.000	0.979	1.000	0.979	1.005	1.000	1.005	1.000	1.000	1.000
Anhui	0.897	0.942	0.952	0.967	1.013	0.954	1.043	1.000	1.043	1.043	1.010	1.033	1.040	1.010	1.030	1.032	1.007	1.025
Fujian	1.007	0.954	1.056	0.943	0.921	1.024	1.011	1.029	0.982	0.973	0.992	0.981	1.015	0.988	1.027	1.008	0.996	1.012
Jiangxi	0.929	0.960	0.968	0.996	1.001	0.996	1.001	1.018	0.983	1.030	0.998	1.032	1.044	1.018	1.026	1.016	1.002	1.014
Shandong	0.900	1.000	0.900	1.131	1.000	1.131	1.115	1.000	1.115	1.010	1.000	1.010	0.998	1.000	0.998	1.035	1.000	1.035
Henan	0.763	0.992	0.769	1.030	0.998	1.032	1.029	1.008	1.021	1.024	1.002	1.023	1.020	1.012	1.008	1.019	1.005	1.014
Hubei	0.919	1.006	0.914	1.022	1.011	1.011	1.031	1.024	1.007	1.052	1.073	0.980	1.038	1.033	1.005	1.017	1.001	1.016
Hunan	0.905	0.945	0.958	1.003	1.007	0.996	1.035	1.026	1.009	1.031	1.012	1.018	1.030	1.005	1.024	1.014	1.000	1.014
Guangdong	1.000	1.000	1.000	1.040	1.000	1.040	1.000	1.000	1.000	1.005	1.000	1.005	1.004	1.000	1.004	1.000	1.000	1.000
Guangxi	1.013	1.018	0.996	1.022	0.995	1.027	1.047	1.044	1.003	0.998	1.119	0.892	0.991	1.006	0.984	1.010	0.998	1.012
Hainan	0.984	1.012	0.972	1.027	1.070	0.960	1.020	1.051	0.970	0.941	1.007	0.935	1.043	1.078	0.968	1.015	1.042	0.974
Chongqing	0.971	1.013	0.958	1.018	1.170	0.870	0.995	0.933	1.066	1.046	1.055	0.991	1.074	1.033	1.040	1.005	1.000	1.005
Sichuan	1.024	0.982	1.042	1.016	0.974	1.043	1.032	0.991	1.041	1.029	1.020	1.009	0.987	0.989	0.998	1.016	1.005	1.011
Guizhou	0.930	0.996	0.933	1.010	1.008	1.003	1.010	1.007	1.003	1.073	1.186	0.905	1.144	1.064	1.075	1.010	1.000	1.009
Yunnan	0.980	1.000	0.980	1.006	1.000	1.006	0.992	1.000	0.992	1.002	1.000	1.002	0.972	1.000	0.972	1.006	1.000	1.006
Sxi	0.938	0.971	0.966	0.984	0.977	1.008	1.018	1.035	0.983	1.006	1.172	0.859	1.024	1.038	0.987	1.003	0.999	1.004
Gansu	1.003	1.004	0.999	1.003	0.999	1.004	1.005	1.020	0.985	1.004	1.001	1.003	1.003	0.999	1.004	1.002	1.000	1.002
Qinghai	0.911	1.000	0.911	0.984	1.000	0.984	0.993	1.000	0.993	1.000	1.000	1.000	1.000	1.000	1.000	1.000	1.000	1.000
Ningxia	0.910	1.000	0.910	1.000	1.000	1.000	1.000	1.000	1.000	1.012	1.000	1.012	1.047	1.000	1.047	1.008	1.000	1.008
Xinjiang	0.957	0.971	0.985	0.980	0.998	0.982	1.016	0.992	1.024	1.018	1.088	0.936	1.061	1.017	1.043	1.001	1.000	1.001
Mean	0.958	0.983	0.976	1.044	1.012	1.033	1.018	1.005	1.013	1.011	1.021	0.991	1.011	1.018	0.993	1.013	1.002	1.011

### Benchmark regression analysis

Before performing spatial regression analysis, this paper uses the forestry industry’s TFP, EC, and TC as the major independent variables by using OLS, fixed effects, random effects and mixed effects models to conduct regression analysis (Table 6). The results show that the impact of TFP on CO_2_ emissions is not a simple linear relationship, but a significant "inverted U" curve, that is, the increase in TFP of the forestry industry in the early stage will promote the increase in CO_2_ emissions, as time goes on, CO_2_ emissions will be reduced in the later stage. TC is similar to TFP, but EC is on the contrary. The estimation coefficients of HGDP are all negative, and except for the fixed effect model, other models passed the significance test with significance level of 10%. This may be due to the influence of positive and negative factors. On the one hand, since the reform and opening up, the rapid growth of China's economy depends on energy consumption to a certain extent. Therefore, economic growth will promote the increase of CO_2_ emissions. On the other hand, with the implementation of the sustainable development strategy, China put energy conservation and emission reduction goals in an important position. Economic growth no longer relies solely on energy consumption, but will drive the development and use of new clean energy through economic development, so as to reduce CO_2_ emissions. According to the results in Table 6 at present, economic development has a more significant inhibitory effect on CO_2_ emissions. Other control variables have no significant effect on CO_2_ emission.

**Table 6:** Benchmark regression results.

Variables	OLS			Fixed effects			Random effects			Mixed effect		
	(1)	(2)	(3)	(4)	(5)	(6)	(7)	(8)	(9)	(10)	(11)	(12)
TFP	2.054			2.604**			2.055*			2.055		
	(1.48)			(1.97)			(1.62)			(1.62)		
TFP^2^	− 4.528**			− 6.440**			− 4.528*			− 4.528*		
	(− 1.93)			(− 2.33)			(− 1.66)			(− 1.66)		
EC		− 1.037			− 1.041			− 1.037			− 1.037	
		(− 0.82)			(− 1.05)			(− 1.13)			(− 1.13)	
EC^2^		1.081			0.945			1.081			1.081	
		(0.38)			(0.26)			(0.32)			(0.32)	
TC			1.844*			1.829**			1.845**			1.845**
			(1.66)			(2.32)			(2.45)			(2.45)
TC^2^			− 3.936			− 3.484*			− 3.936**			− 3.936**
			(− 1.47)			(− 1.71)			(− 2.32)			(− 2.32)
lnURB	1.172	0.560	1.144	− 6.464	− 7.450	− 6.395	1.173	0.560	1.144	1.173	0.560	1.144
	(0.65)	(0.31)	(0.63)	(− 1.03)	(− 1.29)	(− 1.04)	(0.71)	(0.38)	(0.75)	(0.71)	(0.38)	(0.75)
lnFDI	− 0.159	− 0.125	− 0.161	− 0.320	− 0.243	− 0.237	− 0.159	− 0.125	− 0.161	− 0.159	− 0.125	− 0.161
	(− 0.79)	(− 0.62)	(− 0.80)	(− 0.67)	(− 0.50)	(− 0.49)	(− 0.61)	(0.48)	(− 0.63)	(− 0.61)	(0.48)	(− 0.63)
lnHGDP	− 1.567**	− 1.370**	− 1.561**	− 0.859	− 0.454	− 0.721	− 1.567**	− 1.370**	− 1.561**	− 1.567**	− 1.370**	− 1.561**
	(− 2.23)	(− 1.95)	(− 2.21)	(− 0.51)	(− 0.29)	(− 0.44)	(− 2.07)	(− 1.97)	(− 2.18)	(− 2.07)	(− 1.97)	(− 2.18)
lnVOL	0.036	0.034	0.035	− 0.061	− 0.079	− 0.069	0.036	0.024	0.035	− 0.006*	0.034	0.035
	(0.39)	(0.38)	(0.39)	(− 0.55)	(− 0.74)	(− 0.61)	(− 0.38)	(0.37)	(0.37)	(− 1.77)	(0.37)	(0.37)
Obs	300	300	300	300	300	300	300	300	300	300	300	300

***, **and * indicate the significance at 1%, 5% and 10% levels respectively.

### Results of spatial econometrics analysis

#### Testing the spatial effect

As shown in Table 7, China’s CO_2_ emissions exist a significant spatial correlation from 2008 to 2017. The Global $Moran's$* I* of each region are greater than 0, and the Geary's Coefficient (Geary’s C) are less than 1, and they are significant at the 1% statistical level, that is, each region has a significant positive spatial correlation in CO_2_ emissions. However, it can be seen from the coefficient that the spatial correlation gradually decreases with the passage of time.

**Table 7:** The Global $Moran's$ I and Geary's C of CO_2_ emissions.

	MI	Z	C	Z
2008	0.326***	2.195	0.636***	− 2.572
2009	0.310***	2.788	0.660***	− 2.396
2010	0.305***	2.750	0.660***	− 2.391
2011	0.306***	2.736	0.666***	− 2.394
2012	0.285***	2.583	0.675***	− 2.301
2013	0.282***	2.550	0.683***	− 2.259
2014	0.270***	2.475	0.670***	− 2.307
2015	0.270***	2.505	0.664***	− 2.299
2016	0.253***	2.396	0.675***	− 2.174
2017	0.238***	2.249	0.674***	− 2.217

**Figure 6:**
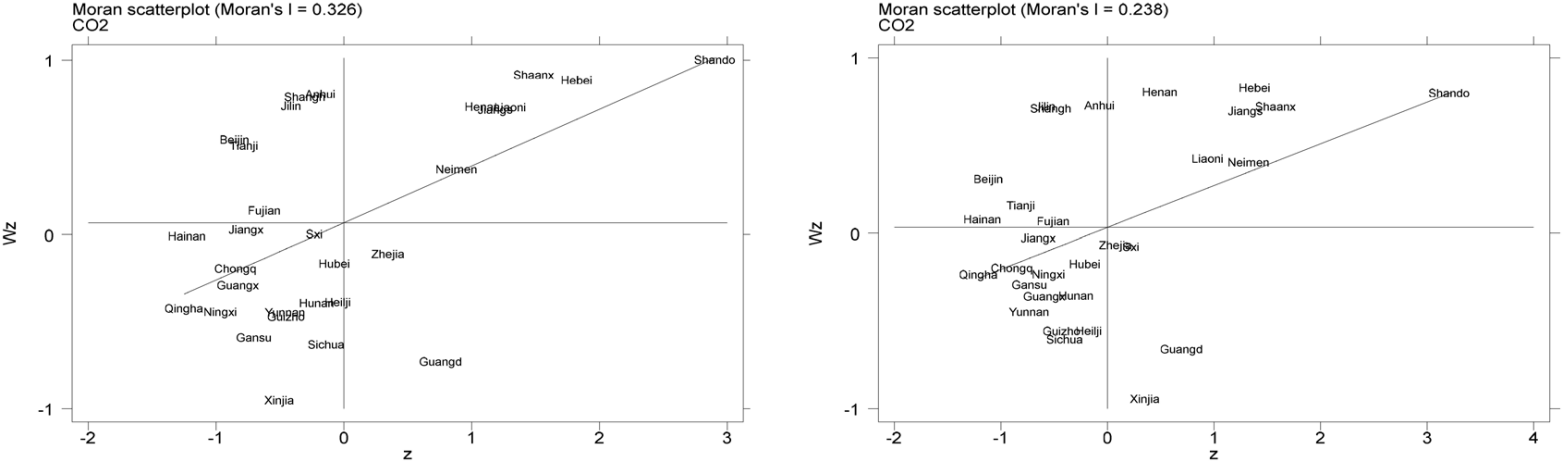
*Moran’s I* scatter diagram of CO2 in 2008 (left) and 2017 (right).

In order to further explore the spatial correlation of CO2 emissions, this paper calculates the local $Moran's$* I* of the CO2 emissions of 30 provinces in China during the sample period, and plots the scatter points in Fig. 6. Obviously, most regions (Heilongjiang, Yunnan, Chongqing, Hebei) are in the first and third quadrants, that is, "high value" is adjacent to the "high value", and the "low value" is adjacent to the "low value", indicating that there is spatial positive correlation. However, Beijing, Shanghai, Fujian, Guangdong, Zhejiang and other places are in the second and fourth quadrants, which means "high value" is adjacent to "low value", and "low value" is adjacent to "high value", showing the negative spatial correlation.

#### Analysis of spatial panel regression results

According to Table 8, the impact of China’s forestry industry’s total factor productivity (TFP) on CO_2_ emissions exhibits an "inverted U-shaped" curve that promotes first and then suppresses. Taking model (16) as an example, the coefficient of the first-order term of TFP is 0.021, the quadratic term is 0.365, and the quadratic term is significant at the 10% statistical level. This is also in line with the Environmental Kuznets (EKC) hypothesis. In addition, compared with the above benchmark regression results, the estimation coefficient with adding spatial factor is smaller. Therefore, ignoring spatial effects will enlarge the impact of forestry industry TFP on CO2 emissions, making the results inaccurate. In model (13)–(15), the estimation coefficient of lnURB is significantly positive at the 1% statistical level, which is 1.736, 2.037, 1.895, respectively, namely urbanization level every 1% increase in CO_2_ emissions increased by 2%. This may be affected by positive and negative factors. On the one hand, the improvement of urbanization level will increase the demand for housing, electricity, automobiles, which will lead to the increase of CO_2_ emissions; on the other hand, the increase of urban population will significantly improve the use efficiency of public goods and reduce CO_2_ emissions. Based on the results of this paper, the urbanization level plays a dominant role in promoting CO_2_ emissions. However, the estimation coefficient of lnFDI is opposite to that of lnurb, both of which are negative, and pass the significance level of 5% significance test. For every 1% increase of FDI, the CO_2_ emission will decrease by about 1%, which shows that the "Pollution Haven Hypothesis" does not conform to the actual situation of China. FDI introduces a large number of talents, funds and technology through "pollution halo metabolism" and technology spillover effect, so as to provide effective support for energy conservation and emission reduction. It is worth mentioning that the estimation coefficient of lnGDP is positive in SAR and SEM models, and negative in SDM model. It also shows that the impact of economic development on CO_2_ emissions is more complex. In the early stage of development, China's economic development relies on energy consumption, and economic growth is also the main driving factor to promote carbon emissions. However, with the development of the concept of "green", the world has recognized the importance of green development, especially China, as a responsible big country, has taken sustainable development as one of the important development goals, gradually slowing down the economic growth, and transforming from a high-yield mode with rapid growth to a high-quality model with sustainable development. From the practical results, China's economic restructuring has achieved the initial goal, the effect of energy conservation and consumption reduction is increasingly apparent, the economic development pays more and more attention to quality and efficiency, and the economic development is changing towards "sound and fast". However, China has a vast territory and abundant resources, and the development of different regions is different. The economy of some regions, including Heilongjiang and Shanxi, still depends on energy consumption to a certain extent, so the inhibition effect of economic development on carbon emissions has not been fully reflected. The estimation coefficients of lnVOL are all negative, showing that the continuous improvement of technology market can promote the progress of energy-saving and emission reduction technology. However, China's technological innovation is more inclined to the innovation of production technology, while the investment in energy-saving and emission reduction technology is relatively small. Therefore, China should promote the progress of green technology, guide the technology research and development of enterprises transfer to "green", and effectively control CO_2_ emissions from perspective of technology.

**Table 8:** Results of the spatial panel data model.

	(13)	(14)	(15)
Variables	SAR	SEM	SDM
W-lnCO2	− 0.276***		− 0.583***
	(− 3.64)		(− 767)
W-u		− 0.524***	
		(− 5. 72)	
TFP	0.104	0.125	0.117
	(1.02)	(1.36)	(1.31)
TFP2	− 0.335*	− 0.401**	− 0.384**
	(− 1.87)	(− 2.31)	(− 2.38)
lnURB	1.736***	2.307***	1.895***
	(4.94)	(6.37)	(5.92)
lnFDI	− 0.071**	− 0.126***	− 0102***
	(− 2.40)	(− 4.15)	(− 3.92)
lnHGDP	0.136	0.049	− 0.015
	(0.80)	(0.32)	(− 0.10)
lnVOL	− 0.005	− 0.003	− 0.003
	(− 0.66)	(− 0.44)	(− 0.44)
W-TFP			0.007
			(0.04)
W-TFP2			0.089*
			(1.68)
W-lnURB			4.561***
			(6.62)
W-lnFDI			− 0.388***
			(− 7.32)
W-HGDP			− 0.291
			(− 1.08)
W-lnVOL			0.013
			(0.82)
R-squared	0.437	0.437	0.457
Log-likelihood	85.481	94.116	122.635
Observation	300	300	300

***, **and * indicate the significance at 1%,5% and 10% levels respectively.

From the perspective of spatial dimensions, the spatial lag coefficients of CO_2_ emissions under the three methods are all significantly negative at the 1% statistical level, once again, it shows that China’s provincial CO_2_ emissions exist obvious spatial agglomeration characteristics. Driven by the energy difference caused by natural resource endowments and the socio-economic activities reflected in the inter-regional industrial structure and product trade, the CO_2_ emissions of a region are closely related to neighboring regions, showing a significant negative correlation. Taking model (10) as an example, a 1% increase in CO_2_ emissions in neighboring areas will cause decreasing of 22.9% in this region. Under the requirements of energy conservation and emission reduction policies, there is a significant "warning effect" between adjacent regions. In the face of the increase of CO_2_ emissions in adjacent regions, the region will pay more attention to energy use and environmental governance, especially the degree of achievement of energy-saving and reduction targets.

According to model (15), firstly, the first and quadratic coefficients of the spatial lag coefficient of forestry industry's TFP (*W-TFP*) are 0.007 and 0.089, and the quadratic coefficient is significantly positive at the 10% statistical level, but not significant at the first order term. This may be due to the different natural resource endowments in different regions, and there are great differences in humidity, soil and tree species. Although affected by the "synergistic effect", there are imitation behaviors among different regions, but the spatial impact is not strong. In the early stage of forestry industry development, due to the limitation of funds, talents and technology, the region will "squeeze" the resources of neighboring areas, and With the increase of national support for forestry, a large number of funds and talents have gathered, the technical level has been significantly improved, and the development of forestry industry in adjacent areas promotes each other.

The spatial lag coefficient (*W-lnURB*) of urb is significantly positive at the level of 1%. The CO_2_ emissions in the region will increase by 4.56% when the urbanization level of adjacent areas increases by 1%. This may be because most of the neighboring regions have similar economic development conditions, under the influence of the “comparison effect” and the “demonstration effect”, the increase in the urban population in the neighboring regions will stimulate the migration of the rural population in the region to urban areas, thus increasing the city’s carbon emissions. The spatial lag coefficient of FDI (*W-lnFDI)* is significantly negative at the 1% significant level, that is, there is a significant negative spatial spillover effect. With the increase of FDI in neighboring regions by 1%, CO_2_ emissions in this region will decrease by 38.8%. This may be due to the increase in the degree of opening up of neighboring areas to the outside world, bringing more foreign-funded enterprises with stricter environmental regulations, which will "force" the improvement of the environmental quality of this region and neighboring regions.

#### Analysis of direct effects, indirect effects and total effects for SDM

This paper further analyzes the impact of forestry industry's total factor productivity (TFP) on CO_2_ emissions through direct effects, indirect effects and total effects. As shown in Table 9, the estimation coefficients of direct effect and total effect of the primary term coefficient of TFP are 0.129 and 0.081 respectively, while the indirect effect is negative, but not significant. The quadratic term coefficient of TFP is opposite to primary term coefficient, indirect effect is positive, direct effect and total effect are negative, and pass significance test of statistical level of 10%. Again, it shows that the impact of TFP of forestry industry on CO_2_ emissions is not a simple linear relationship, but shows an "inverted U-shaped" curve relationship, which is also consistent with the environmental Kuznets hypothesis.

**Table 9:** Results of direct effects, indirect effects and total effects for SDM.

Variables	Direct effect	Indirect effect	Total effect
lnTFP	0.129	− 0.048	0.081
	(1.26)	(− 0.32)	(0.61)
lnTFP2	− 0.434**	0.232	− 0.202*
	(− 2.51)	(0.71)	(− 1.65)
lnURB	1.413***	2.631***	4.044***
	(4.46)	(5.37)	(8.59)
lnFDI	− 0.057**	− 0.252***	− 0.310***
	(− 2.19)	(− 6.63)	(− 8.35)
lnHGDP	0.038***	− 0.206	− 0.167
	(0.24)	(− 0.94)	(− 0.90)
lnVOL	− 0.005	0.012	0.008
	(− 0.69)	(0.98)	(0.59)

From the perspective of control variables, the direct, indirect, and total effect of the estimation coefficients of urbanization level (*lnURB*) are significantly positive at the 1% significance level, indicating that urbanization significantly increases CO_2_ emissions in the region, however, due to the factor, such as "bandwagon effect" and "imitation effect", it will also increase the CO_2_ emissions in adjacent areas. The estimation coefficients of direct, indirect and total effect of FDI are − 0.057, − 0.252, − 0.310, respectively, which have passed the significance test of significance level of 1%. Moreover, every 1% increase of FDI level will reduce CO_2_ emissions of this region by 5.7% and that of neighboring regions by 25.2%. There is no doubt that increasing the level of foreign direct investment will not only help reduce CO_2_ emissions in the region, but also drive foreign direct investment in neighboring regions to identify "green" companies and improve the environment of surrounding area. The estimation coefficient of direct effect of lnHGDP is significantly positive at 1% statistical level, while the estimation coefficient of indirect and total effect is negative, but not significant. The direct effect of lnVOL’s estimation coefficient is negative, the indirect effect and the total effect are positive, but they are not significant. Once again, it shows that China should improve the level of technology market, give certain policy support to technological innovation enterprises, and promote the "green" of technology research and development is significantly positive at 1% statistical level, while the estimation coefficient of indirect effect and total effect is negative, but not significant. The direct effect of lnvol estimation coefficient is negative, the indirect effect and the total effect are positive, but they are not significant. Once again, it shows that China should improve the level of technology market, give certain policy support to technological innovation enterprises, and promote the "green" of technology research and development.

#### Further analysis

Taking the particularity of forestry industry into consideration, this paper recalculates the TFP of China's forestry industry by taking CO_2_ as the unexpected output with other input and output variables remain unchanged. The calculation results show that, from 2008 to 2017, the average TFP of forestry industry with the consideration of unexpected output is 1.007, which is nearly 0.010 higher than the TFP without considering the unexpected output (see Appendix 1). This may be due to the unique nature of the forestry industry itself, the capital and human investment in the early stage of its development may increase the consumption of resources. However, in the later stage of its development, its significant carbon sequestration function may not only reduce the cost of reducing greenhouse gas emissions, but also form a "joint effect" of a large number of forest vegetation accumulation, resulting in "1 + 1 > 2", which can improve the TFP of forestry industry.

At the same time, in order to avoid the multicollinearity of regression results, this paper also takes the new measurement results as the main explanatory variables, and uses the step-by-step method to explore the impact of total factor productivity of forestry industry on CO_2_ emissions from a spatial perspective (see Appendix 2). It should note that except for individual variables, the regression results are similar to the above, which shows the robustness of the results.

**Figure 7:**
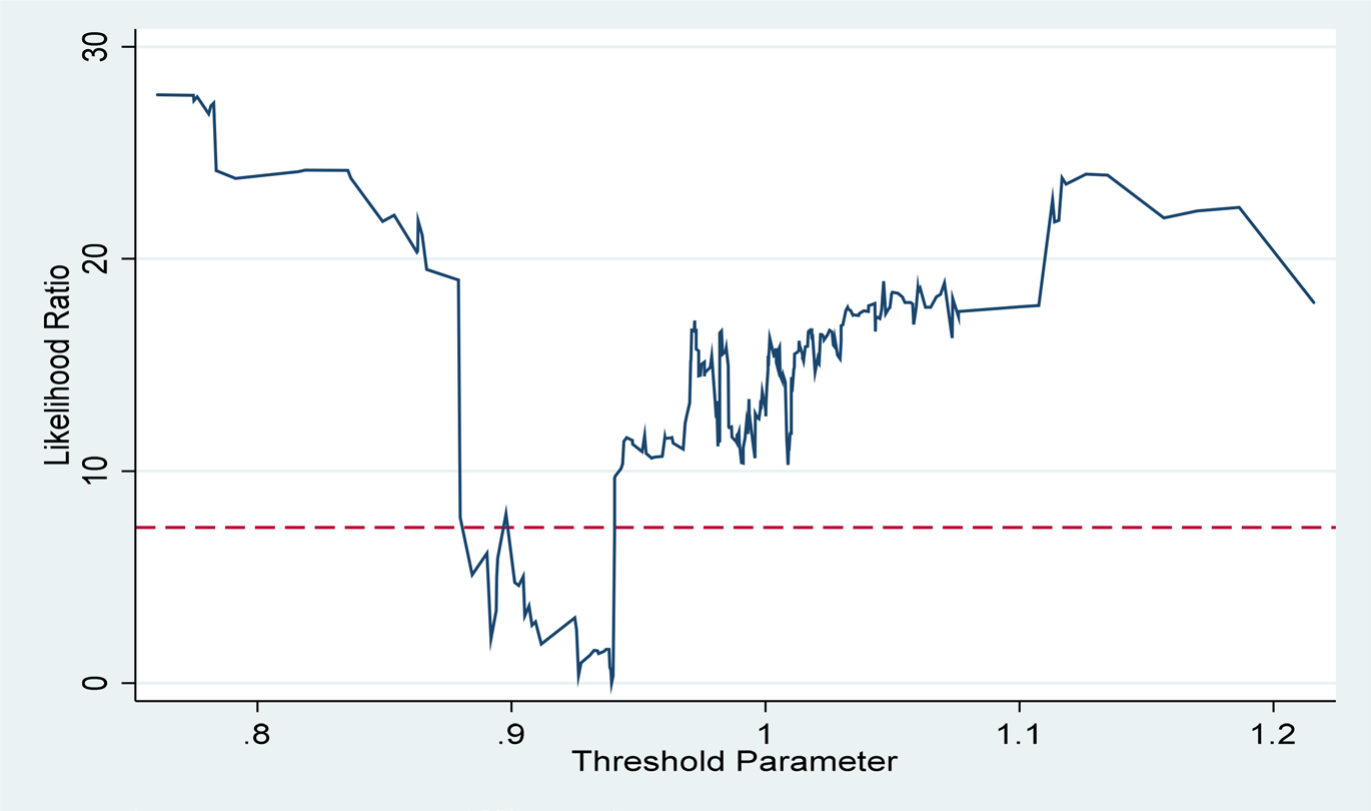
Threshold parameters.

In addition, since the TFP of forestry industry has an “inverted U-shaped” effect on CO_2_ emission, this paper further employs the panel threshold model to find the inflection point of TFP of forestry industry on CO_2_ emission. As shown in Table s 10 and 11, when the TFP of China's forestry industry is greater than 0.9395, it will have an inhibitory effect on CO_2_. Furthermore, Fig. 7 also proves the accuracy of the threshold value.

**Table 10:** Threshold test.

	F value	*P* value	F critical value		
			1%	5%	10%
Single threshold	28.75**	0.023	23.287	25.601	30.377

	Threshold value	95% Confidence Interval			
Single threshold	0.9395	[− 0.017,0.606]

**Table 11:** Threshold effect estimation results.

	(22)
TFP	0.295
(≤ 0.9395)	(0.28)
TFP	− 0.013
(> 0.9395)	(− 1.86)*

### Discussion

According to the above research, we find some interesting phenomena: Firstly, in 2008 to 2017, the development focus of forestry industry gradually shifted from the eastern China to the central and western, which shows the transfer of total factor productivity (TFP) and technical efficiency change (EC) of forestry industry. However, it is worth mentioning that the areas with high efficiency of technical progress (TC) are still mainly concentrated in Shanghai, Jiangsu, Fuzhou and other eastern regions, which is also consistent with the research of Wu and Zhang^39^. In addition to giving full play to its own resource advantages, promoting the technological progress of forestry industry in the central and western regions is also a key factor for its industrial development^20^.

Secondly, the effect of TFP on CO_2_ emission in China's forestry industry shows an inverted U-shaped curve, and the inflection point is 0.9395.At the early stage of the development of the forestry industry, planting trees, reclaiming wasteland, improving forest quality, controlling pests, and increasing soil nutrition require a lot of manpower and material resources, which may cause CO_2_ emissions to rise. After the forestry industry has been fully developed, the total factor productivity reaches above 0.9395, which fully reflects the ecological and economic effects, effectively reduces CO_2_ emissions and promotes economic development^8,11^.

Thirdly, there is a significant negative spatial spillover effect in China's provincial CO_2_ emissions, that is, the increase of CO_2_ emissions in neighboring areas will improve the environmental protection of the region. Under the strategic layout of sustainable development, there is a "warning effect" among regions, especially under the influence of public opinion, environmental department supervision and government officials' performance evaluation, and other factors, the neighboring regions will be taken as "negative cases" to formulate more stringent environmental regulation policies and environmental governance measures, so as to better achieve their own energy conservation and emission reduction goals and reduce CO_2_ emissions. At the same time, it also shows the key considerations for regional joint prevention and control of CO_2_ emissions^21^. It is worth mentioning that the TFP improvement of forestry industry in nearby areas will increase the CO_2_ emission of the region, which requires each region to adjust measures to local conditions and find the forestry production mode suitable for its own development according to its own natural resource endowment, instead of blindly imitating the neighboring regions and limiting the emission reduction work of the region. And the state should increase support for forestry, expand investment, introduce talents, improve the level of technology, alleviate and improve the competition and occupation of forestry resources in neighboring areas at the immature stage of forestry development.

## Conclusions and implications

Accurate identification of CO_2_ emissions is essential for the rational formulation and effective implementation of energy conservation and emission reduction policies, and it is also the key point to promote the green sustainable development. As a key industry to promote the coordinated development of population, economy, society, environment and resources, forestry has a great impact on CO_2_ emissions. Therefore, based on the measurement of CO_2_ emissions, TFP and its decomposition index of 30 provinces in China, this paper constructs the spatial econometric model to empirically study the impact of TFP of forestry industry on CO_2_ emissions from the spatial perspective, and it further analyzes its direct effect, indirect effect and total effect. The major conclusions of the study are as follows: Firstly, the development focus of forestry industry has gradually shifted from the eastern region to the central and western regions from 2008 to 2017, which is mainly reflected in the improvement of total factor productivity (TFP) and technical efficiency change (EC) of forestry industry. Secondly, the impact of TFP of forestry industry on CO_2_ emissions is not a simple linear relationship, but shows an “inverted U-shaped” curve, with the inflection point of 0.9395, and China is now on the right side of the fixed point of the "inverted U-shaped" curve. Thirdly, the spatial spillover effect of CO_2_ emissions is significantly negative because of "warning effect" between adjacent regions. In addition, foreign direct investment, urbanization level, per capita GDP and technology market turnover will also affect regional CO_2_ emissions.

Based on the above conclusions, policy implications for development of forestry industry are provided below:

The first is to strengthen the management of forest resources, and give full play to the pulling function of technological progress on the basis of maintaining the change of technical efficiency and promoting the improvement of total factor productivity. Forest resources are the foundation for the development of forestry industry. Expanding forest area and improving forest management level are the necessary conditions for further development of forestry industry. To be specific, it needs to make effective use of modern forestry technology to strengthen the protection and construction of ecological public welfare forests, such as shelter forests and water conservation forests. At the same time, it also needs to increase the cultivation of commercial forests, such as high-yield forests and precious trees, so as to effectively increase the total amount of forest resources. Moreover, it is crucial to strengthen the protection of forest resources and minimize the occurrence of natural disasters, such as forest degradation, diseases and insect pests^40^.

The second is to develop forestry biomass energy and give full play to the ecological benefits of the industry to make it extend on the right side of the inflection point. The development of forestry biomass energy mainly includes cultivating energy forest and producing energy. It should note that cultivating energy forest species, strengthening the construction of energy forest will give full play to its important role in ecological protection and climate regulation, and then effectively absorbing CO_2_. Furthermore, establishing a forestry biomass energy industry chain with characteristics of energy forest cultivation and biomass energy processing integration will drive the development of new energy forestry, promote the construction of forestry ecological system, and achieve the goal of sustainable development of forestry.

The third is to optimize the forestry industrial structure and improve the economic benefits of the forestry industry. For transforming the development mode of forestry industry, it is crucial to shift the development focus from the primary industry to the tertiary industry, vigorously develop the tertiary industry, scientifically plan the development of tourism service industry in combination with the regional spatial characteristics and resource endowment, and regard it as a new economic growth point to maximize forest advantages and improve economic benefits. While for the secondary industry, it needs to increase the investment in funds and disciplines, improve the quality of scientific research personnel, introduce advanced technology, increase the added value of products, and then turn resource advantages into economic benefits.

Finally, it should strengthen the flow of resources between different regions and form a forestry industry development mode of "rich with poor". Combined with the regression results of the spatial lag item above, if the country desires to improve the growth of forestry industry on the whole, it needs to produce the game competition in forestry production and form the positive interaction of forestry production. Each region should make development plan and strategy according to local conditions and its own characteristics of forestry production factor endowment, and strengthen the spatial interaction influence of forestry production. The flow of production factors can realize the spatial complementarity of factors, improve the spatial allocation efficiency of forestry production factors, and give full play to its role in environmental protection.

## Supplementary Information

Supplementary Information.
