# Supplementary material for: The effect of total factor productivity of forestry industry on CO2 emissions: a spatial econometric analysis of China
Source: Sci Rep. 2021 Jul 9;11:14200. doi: 10.1038/s41598-021-93770-z (PMC8270944; doi:10.1038/s41598-021-93770-z)
Supplement: Supplementary file 1 — Supplementary Information. [file 41598_2021_93770_MOESM1_ESM.docx]

Appendix 1

|  | 2008 | | | 2010 | | | 2012 | | | 2014 | | | 2016 | | | 2017 | | |
| --- | --- | --- | --- | --- | --- | --- | --- | --- | --- | --- | --- | --- | --- | --- | --- | --- | --- | --- |
|  | TFP | EC | TC | TFP | EC | TC | TFP | EC | TC | TFP | EC | TC | TFP | EC | TC | TFP | EC | TC |
| Beijing | 1.011 | 0.978 | 1.034 | 1.028 | 0.961 | 1.069 | 1.024 | 1.024 | 1.000 | 0.961 | 0.948 | 1.014 | 1.032 | 1.023 | 1.008 | 1.027 | 1.025 | 1.002 |
| Tianjin | 0.995 | 0.963 | 1.033 | 0.998 | 0.976 | 1.022 | 0.996 | 0.996 | 1.000 | 1.000 | 0.999 | 1.001 | 0.999 | 1.088 | 0.918 | 1.002 | 1.000 | 1.002 |
| Hebei | 0.879 | 0.966 | 0.910 | 1.009 | 0.959 | 1.052 | 1.022 | 1.027 | 0.994 | 1.010 | 1.020 | 0.991 | 1.006 | 1.142 | 0.881 | 1.017 | 1.001 | 1.016 |
| Shaanxi | 0.866 | 0.998 | 0.867 | 1.018 | 1.040 | 0.979 | 1.001 | 0.998 | 1.003 | 1.008 | 1.003 | 1.005 | 1.012 | 1.153 | 0.878 | 1.010 | 1.000 | 1.010 |
| Neimeng | 0.953 | 1.000 | 0.953 | 0.974 | 1.000 | 0.974 | 0.953 | 0.982 | 0.971 | 0.924 | 1.022 | 0.904 | 1.001 | 1.006 | 0.995 | 0.990 | 1.000 | 0.990 |
| Liaoning | 0.876 | 0.928 | 0.944 | 1.018 | 0.998 | 1.020 | 1.011 | 0.991 | 1.020 | 0.991 | 0.973 | 1.018 | 0.999 | 1.033 | 0.967 | 1.003 | 1.001 | 1.002 |
| Jilin | 1.005 | 1.074 | 0.936 | 1.028 | 1.025 | 1.003 | 0.988 | 0.997 | 0.991 | 1.021 | 1.021 | 1.000 | 1.009 | 0.981 | 1.029 | 1.013 | 1.006 | 1.007 |
| Heiljiang | 0.940 | 0.993 | 0.947 | 1.007 | 0.994 | 1.013 | 0.994 | 1.025 | 0.970 | 1.024 | 1.041 | 0.983 | 0.998 | 0.958 | 1.042 | 0.997 | 1.001 | 0.996 |
| Shanghai | 0.963 | 1.000 | 0.963 | 0.979 | 1.000 | 0.979 | 1.000 | 1.000 | 1.000 | 1.030 | 1.000 | 1.030 | 0.821 | 1.000 | 0.821 | 1.033 | 1.000 | 1.033 |
| Jiangsu | 1.068 | 1.000 | 1.068 | 1.181 | 1.000 | 1.181 | 1.018 | 1.000 | 1.018 | 1.022 | 0.989 | 1.034 | 1.012 | 1.000 | 1.012 | 1.023 | 1.000 | 1.023 |
| Zhejiang | 1.000 | 1.000 | 1.000 | 1.003 | 1.000 | 1.003 | 1.002 | 1.000 | 1.002 | 0.978 | 1.000 | 0.978 | 1.005 | 1.000 | 1.005 | 1.000 | 1.000 | 1.000 |
| Anhui | 0.911 | 0.996 | 0.915 | 1.023 | 1.064 | 0.962 | 1.073 | 1.036 | 1.035 | 1.033 | 0.975 | 1.059 | 1.066 | 1.056 | 1.009 | 1.049 | 1.021 | 1.028 |
| Fujian | 0.992 | 1.000 | 0.992 | 0.995 | 1.000 | 0.995 | 0.990 | 1.000 | 0.990 | 1.013 | 1.000 | 1.013 | 1.019 | 1.000 | 1.019 | 1.018 | 1.000 | 1.018 |
| Jiangxi | 0.942 | 1.000 | 0.942 | 1.012 | 1.000 | 1.012 | 1.002 | 1.000 | 1.002 | 1.007 | 1.000 | 1.007 | 1.033 | 1.000 | 1.033 | 1.011 | 1.000 | 1.011 |
| Shandong | 0.912 | 1.000 | 0.912 | 1.155 | 1.000 | 1.155 | 1.094 | 1.000 | 1.094 | 1.029 | 1.000 | 1.029 | 0.990 | 1.000 | 0.990 | 1.010 | 1.000 | 1.010 |
| Henan | 0.791 | 0.987 | 0.802 | 1.023 | 0.948 | 1.079 | 1.013 | 1.004 | 1.008 | 1.023 | 1.002 | 1.021 | 1.017 | 1.006 | 1.011 | 1.021 | 1.014 | 1.007 |
| Hubei | 0.898 | 1.007 | 0.892 | 1.013 | 0.986 | 1.028 | 1.033 | 1.019 | 1.014 | 1.116 | 1.113 | 1.003 | 1.058 | 1.038 | 1.020 | 1.023 | 1.008 | 1.015 |
| Hunan | 0.880 | 0.959 | 0.918 | 0.976 | 0.961 | 1.016 | 1.029 | 1.034 | 0.995 | 1.073 | 1.037 | 1.034 | 1.039 | 1.000 | 1.039 | 1.023 | 1.000 | 1.023 |
| Guangdong | 0.996 | 1.000 | 0.996 | 1.023 | 1.000 | 1.023 | 1.000 | 1.000 | 1.000 | 1.002 | 1.000 | 1.002 | 1.005 | 1.000 | 1.005 | 1.000 | 1.000 | 1.000 |
| Guangxi | 0.980 | 1.088 | 0.900 | 1.002 | 1.000 | 1.002 | 0.986 | 1.000 | 0.986 | 1.031 | 1.000 | 1.031 | 1.027 | 1.000 | 1.027 | 1.000 | 1.000 | 1.000 |
| Hainan | 0.799 | 0.968 | 0.826 | 1.001 | 0.978 | 1.023 | 0.980 | 0.963 | 1.018 | 1.045 | 1.023 | 1.022 | 0.992 | 0.985 | 1.008 | 1.016 | 1.001 | 1.014 |
| Chongqing | 0.996 | 1.027 | 0.970 | 1.006 | 1.127 | 0.893 | 0.991 | 0.934 | 1.061 | 1.086 | 1.095 | 0.992 | 1.073 | 1.000 | 1.073 | 1.017 | 1.000 | 1.017 |
| Sichuan | 0.984 | 1.061 | 0.927 | 0.992 | 1.001 | 0.991 | 1.023 | 1.017 | 1.006 | 1.021 | 1.012 | 1.009 | 1.033 | 1.001 | 1.032 | 1.033 | 1.009 | 1.025 |
| Guizhou | 0.933 | 0.981 | 0.951 | 0.990 | 0.974 | 1.016 | 0.998 | 1.017 | 0.981 | 1.029 | 1.025 | 1.004 | 1.214 | 1.157 | 1.049 | 1.002 | 0.988 | 1.014 |
| Yunnan | 0.961 | 1.000 | 0.961 | 0.974 | 1.000 | 0.974 | 0.987 | 1.000 | 0.987 | 1.020 | 1.039 | 0.982 | 1.035 | 1.000 | 1.035 | 1.011 | 1.000 | 1.011 |
| Sxi | 0.922 | 0.975 | 0.945 | 0.989 | 1.008 | 0.980 | 1.000 | 1.009 | 0.991 | 1.005 | 1.008 | 0.998 | 1.015 | 1.074 | 0.945 | 0.999 | 0.997 | 1.002 |
| Gansu | 0.983 | 1.021 | 0.963 | 1.010 | 1.014 | 0.996 | 0.976 | 1.004 | 0.973 | 1.000 | 1.015 | 0.985 | 1.011 | 1.069 | 0.946 | 1.013 | 1.012 | 1.001 |
| Qinghai | 0.966 | 1.000 | 0.966 | 0.992 | 1.000 | 0.992 | 0.958 | 1.000 | 0.958 | 1.000 | 1.000 | 1.000 | 1.003 | 1.000 | 1.003 | 0.991 | 1.000 | 0.991 |
| Ningxia | 0.965 | 1.000 | 0.965 | 1.000 | 1.000 | 1.000 | 1.000 | 1.000 | 1.000 | 1.011 | 1.000 | 1.011 | 1.045 | 1.000 | 1.045 | 1.007 | 1.000 | 1.007 |
| Xinjiang | 1.003 | 1.061 | 0.946 | 0.973 | 1.071 | 0.908 | 0.983 | 0.982 | 1.001 | 1.007 | 1.007 | 1.000 | 1.092 | 1.029 | 1.061 | 1.001 | 1.000 | 1.001 |
| Mean | 0.946 | 1.001 | 0.945 | 1.013 | 1.003 | 1.011 | 1.004 | 1.002 | 1.002 | 1.017 | 1.012 | 1.005 | 1.022 | 1.027 | 0.997 | 1.012 | 1.003 | 0.946 |

Appendix 2

| SAR | (16) | (17) | (18) | (19) | (20) | (21) |
| --- | --- | --- | --- | --- | --- | --- |
| W-lnCO2 | -0.129* | -0.126* | -0.226*** | -0.257*** | -0.257*** | -0.257*** |
|  | (-1.72) | (-1.68) | (-3.01) | (-3.38) | (-3.39) | (-3.39) |
| TFP | 0.055 | 0.085 | 0.034 | 0.019 | 0.025 | 0.024 |
|  | (0.70) | (0.95) | (0.40) | (0.22) | (0.29) | (0.28) |
| TFP2 |  | -0.131* | -0.052* | -0.027* | -0.019* | -0.012* |
|  |  | (-1.69) | (-1.59) | (-1.51) | (-1.71) | (-1.78) |
| lnURB |  |  | 0.848*** | 0.893*** | 0.846*** | 0.838*** |
|  |  |  | (5.31) | (5.59) | (4.74) | (4.69) |
| lnFDI |  |  |  | -0.032** | -0.032** | -0.033** |
|  |  |  |  | (-2.17) | (-2.17) | (-2.19) |
| lnHGDP |  |  |  |  |  | 0.051 |
|  |  |  |  |  |  | (0.59) |
| lnVOL |  |  |  |  |  | 0.057 |
|  |  |  |  |  |  | (0.65) |
